# Impacts of Dam Age on Lifetime Productivity of Angus Replacement Beef Females

**DOI:** 10.3390/ani12202768

**Published:** 2022-10-14

**Authors:** Krista R. Wellnitz, Cory T. Parsons, Julia M. Dafoe, Darrin L. Boss, Samuel A. Wyffels, Timothy DelCurto, Megan L. Van Emon

**Affiliations:** 1 Department of Animal and Range Sciences, Montana State University, Bozeman, MT 59717, USA; 2 Northern Agricultural Research Center, Montana State University, Havre, MT 59501, USA

**Keywords:** dam age, lifetime productivity, replacement heifer

## Abstract

**Simple Summary:**

Selecting replacement beef females is often based on individual producers and their own individual needs. Selection criteria typically vary from producer to producer but generally include productivity of the mother, ability to rebreed and wean a heavy calf. Increased longevity of the dam may be an indicator of potential productivity of their female offspring. Greater longevity of females with beef herds allows producers to be more selective when choosing replacement females; although, this may increase generation intervals. Using comprehensive cow-calf production data can help improve overall selection criteria associated with cow-calf productivity such as reproductive ability, weaning weights of offspring and ability to remain in the herd.

**Abstract:**

Comprehensive cow-calf production data was utilized to evaluate the impact of dam age on lifetime productivity of Angus replacement beef females. Cows used in this study were commercial Angus replacement females born between 2006 and 2020, ranging in age from 1 to 14-yr of age (*n* = 3568). To determine the impact of dam age on lifetime productivity, cows were classified by age groups, specifically 2-, 3-, 4-, 5-, 6/7-, and 8-yrs old and older. The 8-yr and older group consisted of females that were up to 14-yr of age. Cow BW at breeding exhibited a cow age × dam age interaction (*p* < 0.01). Cows at 5-yrs of age from 2-yr old dams weighed less at breeding than cows at 5-yrs of age from 3-, 4-, 5- and 8-years and older dams, with cows at 5-yr of age from 6/7-yr old dams being intermediate. The probability of remaining in the herd at the age of 5 was significant for dam age (*p* = 0.05) averaging 69.41%, but after further delineation, no significant impacts of dam age were observed. Productivity as a measure of total pounds of calf weaned through 5-yrs displayed a dam age effect (*p* = 0.01) with cows from 8-yrs or older dams weaning more total pounds of calf, than cows from 3-yr-old dams. In summary, the impact of dam age on lifetime productivity indicates that dam age can impact future productivity of their offspring.

## 1. Introduction

Lifetime productivity, typically measured as the total weight of calves weaned during a cow’s lifetime, is one of the most important components of efficiency in beef cow-calf production because it is a function of survival and reproductive performance of cows and of survival and growth rate of their offspring [1]. Additionally, cows that remain in a herd longer reduce the cost of replacements on a yearly basis. Production and reproductive traits are a function of fertility, maternal ability and survival of the cows, as well as, of prenatal and postnatal survival and preweaning growth of their offspring. Average cow age (generational interval) in the producing cow herd is a function of when a cow is no longer productive due to the loss of calf, poor calf performance, illness, death, infertility, and/or unsoundness [2,3].

For a herd to be profitable, it is necessary for first-calf and mature cows to breed in a timely manner to ensure a 365-d calving interval. In order to ensure maximum potential for lifetime productivity, researchers suggest first-calf heifers calving between 23 and 25 months of age [4]. In relation, these females should remain in the herd long enough to pay for their development and maintenance cost [5,6]. The number of cows remaining in production past this breakeven age, typically 5-yrs in beef herds, must compensate for those cows that are culled before that age [5]. Greater female longevity/dam age with the herdallows producers to be more selective when choosing replacement females [7]. Longevity traits reflect the performance of a cow over her total herd life and can only be measured after the cow has been culled [8]. An alternative measure of herd life is the use of stayability traits. Stayability, as a measure of herd life, is defined as the probability of the dam surviving to a specific age, given the opportunity to reach that age [9].

Selection of replacement heifers plays a key role in productivity of beef cow herds as well. When selecting replacement heifers, it is important that they are selected under the same management and environmental settings that their offspring will be raised in order to understand the individual animal fit within the environment that they are expected to function [10]. Several studies have shown the relationship between calving early in the calving season and increased longevity in cattle [11,12,13]. Typically, heifers born earlier in the calving season are more likely to be retained as they have an increased likelihood of being greater in size and capacity then their younger counterparts at selection [14]. In addition, heifers born to cows that have had one or more calves have shown increased pregnancy rates in their second breeding season when compared to first-calf heifers [15]. Furthermore, early born calves are also born to dams that were fertile at the beginning of the breeding season [16] and, as a result, likely are more suited to the reproductive environment.

Herd structure, based on individual management decisions related to culling requires considerable discussion. Maintaining cows in a herd for a shorter period of time requires increased replacement heifers, therefore shifting the median age to younger animals. It is well understood that younger/smaller females will require less feed resources than older females, but also potentially sacrifice total calf weaned compared to older more mature females [16].

Over time, using extensive record-keeping information allows cow-calf producers to make more informed selection decisions based on cattle productivity. Tracking aspects of individual animal productivity over time can allow beef cow-calf producers to select efficient and productive female offspring for the environment in which they are managed. Although research utilizing extensive records has evaluated metrics related to the longevity of beef females in the herd, including reproductive efficiency [4,8,14,17] nutritional management to ensure adequate size and weight for breeding [8,17] information related to the effects of dam age on female offspring longevity in the herd is minimal [18]. Dam age could be a meaningful metric for managing cattle longevity, as cattle that remain in a herd past their breakeven age likely fit the environment they are managed in, which may be passed on to their female offspring. Therefore, the objectives of this study were to determine the impacts of dam age on the lifetime productivity of their female offspring.

## 2. Materials and Methods

### 2.1. Cow—Calf Production Data

Comprehensive cow and calf performance data has been collected at Northern Agricultural Research Center, Havre, MT, since 2005. Cows used in this study are commercial Angus females (*n* = 3568; Supplementary Table S1) born and raised at the Northern Agricultural Research Center (NARC; Havre, MT, USA; 48.5500° N, 109.6841° W) between 2006 and 2020, ranging in age from 1 to 14-yr of age. To determine the impact of dam age on lifetime productivity, progeny and yearling heifer data were used. In addition, bred cows were classified by age group, specifically 2-, 3-, 4-, 5-, 6/7-, and 8-yr old and older to determine the impact of dam age on lifetime productivity. The 8-yr and older group consisted of females 8 and 14-yr of age. The 6 and 7-yr-old cows were combined to ensure group sizes were similar. Individual animal data is collected on all NARC females throughout their lifetime. Individual animal data includes calf Julian birth date, calf birth weight, calf weaning weight (WW), calf 205-d weight, calf yearling weight (YW), weaning weight ratio (WWR), cow body weight (BW) at weaning and breeding, and cow body condition scores (BCS). As females are bred, lifetime productivity data is collected each year, specifically reproductive data including pregnancy status, conception percentage and whether or not females were bred via artificial insemination or natural service. This data is collected starting at birth and ends when an animal is culled from the herd. All females that are culled from the herd are recorded for cause of culling which may include pregnancy status (open or out of normal breeding season), disposition concerns, or displayed structural concerns (lameness, teeth, feet/legs, udder). In addition, beginning in 2011, females that did not conceive by artificial insemination did not remain in the herd. For clarification, dam age data should be thought of as the data related to the mother of the calf, while cow age is related to the females as they become productive bred females within the herd.

### 2.2. Statistical Analysis

The data and number of observations in each age category used in this manuscript are available in the Supplementary Lifetime Productivity Tables file. Beef cow production and reproduction data parameters included calf related data, specifically calf birth weight, calf WW, calf 205-d weight, weaning weight ratio (WWR) and calf Julian birth date. Cow specific production and reproduction parameters included cow yearling weight, cow weight at breeding, cow BCS at weaning, cow weight at weaning, cow BCS at weaning, cow years in the herd, productivity through 5-yrs. Production and reproduction characteristics were analyzed using ANOVA with a mixed model that included cow age, dam age, and the interaction of cow age and dam age as fixed effects, and individual cow as the random effect (lme4; [19]). In addition, AI conception rate and pregnancy status were analyzed using generalized linear models following a binomial distribution in an ANOVA framework (car; glm [20]). Individual animal was considered the experimental unit; specifically, each animal was used from the time they were born through culling. Data were plotted and transformed if needed to satisfy assumptions of normality and homogeneity of variance. An alpha ≤0.10 was considered significant. The Tukey method was used to separate means when alpha was <0.05 (emmeans; [21]). All statistical analyses were performed in R [22].

## 3. Results

The effect of dam age on subsequent production measures and cow longevity of Angus beef females is detailed in Table 1 and Table 2. Cow BW at breeding exhibited a cow × dam age interaction (*p* < 0.01). Cows at 5-yrs of age born from 2-yr old dams weighed less at breeding than cows at 5-yrs of age born from 3-, 4-, 5- and 8-yrs and older dams, with cows at 5-yr of age born from 6/7-yr old dams being intermediate. There were no significant cow age × dam age interactions (*p* ≥ 0.16) for lifetime productivity variables. Therefore, only main effects will be presented for all other variables.

### 3.1. Dam Age Effects

Cow BW at weaning displayed significance for dam age (*p* = 0.04) with cows born from 5- and 8-yr old and older dams having greater BW than cows born from 2-yr old dams with 3-, 4-, and 6/7-yr old dams being intermediate. Calculated calf 205-d weights displayed an effect of dam age (*p* = 0.03) with cows born from 2- and 3-yr old dams producing calves with greater 205-d weights than cows born from 6/7- and 8-yr old and older dams, while cows born from 3- and 4-yr old dams were intermediate. Weaning weight ratio (WWR) was significant for dam age (*p* = 0.10) with cows born from 2- and 3-yr old dams having the greatest WWR. Pregnancy status was also significant for dam age (*p* = 0.08) with cows from 8-yr old dams having greater pregnancy rates than all other age classes. There was no effect (*p* ≥ 0.19) of dam age on BCS at weaning, calf birth weight, calf weaning weight, calf Julian birth date or AI conception.

Cow yearling weight (Table 2) was significant for dam age (*p* < 0.01) with cows from 5-, 6/7-, and 8-years and older dams having greater yearling weights than cows from 2- and 3-yr old dams, with cows from 4-yr old dams being intermediate. The probability of remaining in the herd at 5-yr old was significant (*p* = 0.05), but upon further delineation, there were no significant impacts from dam age. The probability of remaining in the herd until the age of 5 averaged 69.41% across all age groups. Productivity as a measure of total pounds of calf weaned through 5 yrs displayed a dam age effect (*p* = 0.01) with cows from 8-yrs or older dams weaning more total pounds of calf than cows from 3-yr old dams. All other age groups were intermediate.

### 3.2. Cow Age Effects

The influence of cow age on beef cow production and reproductive measurements for Angus beef females is detailed in Table 3. Cow BCS were significant for cow age (*p* < 0.01) with BCS increasing from 2 through 7-yrs of age before decreasing in cows that were 8-yr or older. Cow weight at weaning displayed an effect of cow age (*p* < 0.01) with weights increasing from 2 through 7 yrs of age before declining in cows that were 8-yrs of age or older. As expected, calf birth weights were significant for cow age (*p* < 0.01) with 2- and 3-yr old cows having lighter offspring, while 6–7- and 8-yr old cows had the heaviest calves, while 4- and 5-yr old cows being intermediate. In addition, calf 205-d weights displayed an effect of cow age (*p* < 0.01) with 205-d weights increasing from 2 through 4 years of age, with 6/7-yr old cows having the heaviest weights, while 5- and 8- and older cows were intermediate. Calf weaning weights displayed an effect of age (*p* < 0.01) with increasing weights from 2 through 4 years of age, while 6/7- and 8 and older cows had the heaviest offspring with 5-yr old cows being intermediate.

## 4. Discussion

Cow longevity is a major factor to consider in commercial cow-calf production. Increased longevity decreases the need for increased numbers of replacement heifers. Although there is a potential genetic advantage to first-calf heifers, there is also a significant cost associated with raising replacement heifers. Conventional management practices suggest that a majority of beef producers will sell progeny from younger females; typically from females that are 2–3 yrs of age, as they believe they will never perform as well as their contemporaries [23]. The age to which individual cows remain in the herd varies based on individual management decisions and needs of the operation. Multiple studies have determined varying ranges of longevity within beef herds across the United States. Research by Tanida and colleagues [2] compared two different beef herds (Hereford and Angus) over 30 yrs and 23 yrs, respectively. It should be noted that the average first calving of the Hereford herd was 3.2-yr of age, while the average age of the Angus females at first calving was 2-yr of age. Furthermore, females from the Hereford herd were typically culled at 10-yr of age, while the Angus herd generally culled based on non-pregnancy, poor production, calving difficulty and poor maternal ability. Results indicated that the average longevity of over time was 4.21 ± 0.06 yrs for Hereford and 4.49 ± 0.13 yrs for Angus from first calving to removal from the herd and 7.40 ± 0.06 and 6.68 ± 0.12 yr from birth to removal from the herd, respectively. The lack of differences in production results between the two herds is potentially due to differences in sire, culling criteria, and overall management practices. In relation, Stewart and Martin [24] analyzed performance data from Angus cows over a 12-yr period and indicated that the average herd life was 7.4 yrs. Although not indicated in the results, we calculated longevity within our Angus herd to be 7.24 yrs which was similar to Stewart and Martin [24]. Brigham and coworkers [25] reported that cow longevity and overall productivity was greater for females that were at least four years of age and have weaned at least three calves. Longevity in our study was determined as the average years in the herd for animals that were yearlings between 2006 and 2014; specifically, animals that had the opportunity to have four calves and were bred for the fifth.

Although cow longevity and overall herd dynamics is going to vary from herd to herd, one of the major factors influencing this production process is reproductive success of all beef females, first-calf heifers and mature cows. According to National Animal Health Monitoring System (NAHMS) data [26] one-third of all cows are culled from the herd due to reproductive failure. A study by Etienne and Martin [27] utilized 144 Angus females and determined that 60.4% of females were culled from the herd due to reproductive failure. Other reasons that females were culled from the herd included body condition or age, no calf at their side, death or physical/structural issues. Data from Boyer and colleagues [28] indicated that in order for replacement females to cover their developmental costs and maintenance expenses they must have at least six calves. Data from Clark and coworkers [29] reported slightly lower numbers, suggesting 3 to 5 calves in order for a heifer to repay her development costs. Based on the information provided in the previously mentioned papers, we calculated longevity as the average years in the herd for animals that had the opportunity to have four calves and were bred for the fifth, therefore meeting the baseline for repaying development costs. Either way it is well understood that reproductively sound females are more likely to remain in the herd longer than those that do not rebreed in a timely manner. Data from Cushman and colleagues [30] indicated that heifers from the United States Meat Animal Research Center (USMARC) beef herd that calved within the first 21-d of their first calving season had a significantly better chance of remaining in the herd through their fifth calf than females that calved from 23 d to greater than 43 d. This data suggests that females that calve early in the calving season, produce ≥5 calves over a six-year period and have the ability to rebreed in a timely manner, have proven reproductive performance. Beef females that have the ability to maintain physical and reproductive soundness in limited nutritional environments while weaning a heavy calf over their productive lifetime provide an economic advantage to beef producers.

A recent study by Beard and colleagues [18] investigated the impacts of cow age on heifer progeny performance and longevity from 1059 Husker red cows that ranged from 2 to 11 yrs of age. Their data indicated that heifer calves born to younger (2 to 3 yrs old) cows had lighter birth weights and 205-d weights than those born to moderate (4 to 6 yrs old) and old (≥7 yrs old) cows. However, in our study, cows born to young dams (2- and 3-yrs old) had greater 205-d weights than their older counterparts, which was not consistent with their productivity through 5-yr. This may be due to the fact that the younger females were partitioning the extra energy to milk production, while the more mature females partitioned extra nutrients to body condition. The inconsistency may be due, in part, by the 205-d weight being an adjusted weight to 205-d of age, whereas the productivity through 5-yrs is a summation of the actual weaning weight within age group. Interestingly, Beard and coworkers [18] did determine that number of calf crops from young dams was greater when compared to moderate and old cows. However, this in part, may be due to the number of cows in the young category compared with the moderate and old cows, which was not reported.

Additional research out of Nebraska by da Silva and coworkers [15] evaluated the effects of dam age on female calf productivity through her second breeding season. Their data indicated that as dam age increased, heifer adjusted 205-d BW increased until 7 to 8 yrs of age. This data is in agreement with Funston and Deutscher [17] who suggested that increased weights prior to breeding were due to increased milk and overall nutrient availability to those earlier born calves compared to their later born contemporaries. In addition, pre-breeding BW were greater in heifer calves born to older dams than younger dams. Data also was significant for dam age to influence the percentage of heifers that were pre-pubertal prior to breeding season with heifers born to older dams having greater cyclicity rates than heifers born to young dams. In the current study, there was an increase in pregnancy status in cows from 8-yr and older dams. Pregnancy status or the ability of the females to rebreed in a timely manner is one of the biggest, if not the biggest determinant of a female remaining in the herd. In relation, our data is in agreement with da Silva and coworkers [15], where calf 205-d weights were greater in calves born to older cows than younger cows which is consistent with our findings. Contradictory to data from da Silva and coworkers, our data displayed an increase in pregnancy status in offspring born to8-yr old cows compared to other age classes.

In relation to previously mentioned studies, Cundiff and coworkers [1], determined that WW per cow exposed increased as cow age increased from 2 to 5-yrs of age, peaked from 5 to 9-yrs of age and declined from 9 to 12-yrs of age. This data is in accordance with our data, where WW values increased in offspring through 7-yrs before declining at 8-yrs and older. This suggests that cow age directly impacts calf WW, but the age of the cow’s dam has minimal impacts, with only the younger cows (≤4-yrs old) to have greater WWR than older cows (≥5-yrs old). Not only are median aged cows weaning heavier calves than younger and more senior cows, their WWR are greater as well.

Although not discussed in this paper, it is also important to consider the differences that may occur in calves due to breed differences. Tanida and colleagues [2] reported the number of calves weaned in Hereford cows was 3.46 calves and 3.66 for Angus cows over 30 yrs and 23 yrs, respectively. Meanwhile, Stewart and Martin [24], reported total number of weaned calves to be 6.4 over a 12-yr period. Therefore, our results may be breed specific and may be one of the main reasons that we see the differences observed between the current data and previous research.

Stewart and Martin [24] reported the total amount of calf weight weaned for 113 Angus cows over 12-yr was 1283 kg respectively. Our research indicates an average of 683.0 kg of total calf weaned for dams from 2 through 8-yr old and older. These differences are likely due to the increase in data available for our study compared to Stewart and Martin, respectively. In addition, cow size may have been different between studies. Furthermore, there was no effect of cow age or dam age on calf Julian birth date or AI conception percentage. Overall, these previously mentioned studies indicate that older cows may have a positive impact on growth and productivity of female offspring.

## 5. Conclusions

Data from our study suggest that dam age impacts the future outcomes of replacement heifers based on productivity and reproductive measures. In general, offspring born to dams that are of moderate age (≥5 or older) will have increased productivity compared to those born to younger animals (≤4 or younger). Likely this is going to be due to the fact that these females being retained are able to rebreed in a timely manner, while maintaining milk production and physical structure in order to allow them to raise a healthy calf to weaning This data suggests that producers might want to consider the cost–benefit ratio of raising a large number of replacement heifers compared to retaining the older, reproductively sound and proven mature females instead. Economically, according to our data, selecting mature females over first-calf heifers allows for decreased input costs associated with raising a large number of replacement heifers. In other words, offspring born to older dams, which are raised in similar environments, which are characteristically associated with high-fiber, low quality and low levels of yearly precipitation allows producers to make selection decisions based on multiple criteria. Producers have the potential to decrease costs associated with raising a large number of replacements heifers due in part to the fact that the mature cows are continuing to be productive for a longer period of time. However, further research is needed to fully understand the impact of dam age on lifetime productivity of female offspring. Specifically, a better understanding of the value of mature, proven females and their success in limited nutrition forage-based beef production systems and the overall economic impact on cow-calf profitability.

## Figures and Tables

**Table 1:** The effect of dam age on subsequent production measures and cow longevity in Angus beef females.

	Dam Age, Years							*p*-Value		
Category	2	3	4	5	6/7 ^4^	8+ ^5^	SE ^1^	Cow Age	Dam Age	Cow × Dam
Cows, *n*	733	590	575	427	433	810				
Cow age; wt. at breeding, kg								<0.01	0.27	<0.01
2 yrs.	471.0	486.66	489.90	496.52	497.81	477.34	13.85			
3 yrs.	524.66	520.92	533.82	550.52	522.08	519.96	14.11			
4 yrs.	567.02	586.36	580.25	606.73	581.25	563.72	14.13			
5 yrs.	557.64 ^a^	609.65 ^b^	612.53 ^b^	627.70 ^b^	584.70 ^ab^	609.08 ^b^	15.48			
6/7 yrs.	611.88	621.04	626.35	619.28	643.99	635.20	14.48			
8+ yrs.	627.23	628.54	601.62	600.75	619.70	631.72	15.24			
Cow BCS at weaning	5.34	5.32	5.32	5.37	5.33	5.33	0.09	<0.01	0.34	0.19
Cow wt. at weaning, kg	598.50 ^a^	617.63 ^ab^	617.55 ^ab^	627.94 ^b^	619.72 ^ab^	620.39 ^b^	6.97	<0.01	0.04	0.16
Calf birth wt., kg	39.55	40.85	40.63	39.39	39.60	39.89	0.63	<0.01	0.19	0.31
Calf 205 d wt., kg	270.07 ^a^	271.46 ^a^	263.32 ^ab^	265.46 ^ab^	257.06 ^b^	261.43 ^b^	5.39	<0.01	0.03	0.29
Calf weaning wt ^2^, kg	254.37	256.30	249.65	252.52	244.04	247.48	5.12	<0.01	0.24	0.91
Calf Julian birth date	80.97	81.12	80.79	80.07	81.26	81.36	1.74	0.12	0.49	0.81
Weaning weight ratio ^3^, %	42.49	42.07	40.72	40.32	40.22	40.26	1.02	0.56	0.10	0.63
Pregnancy status, %	88.13	87.16	88.03	88.65	87.95	90.14	1.68	0.44	0.08	0.45
AI conception, %	62.76	59.68	64.42	63.07	64.00	59.67	2.91	0.98	0.85	0.98

^1^ Pooled standard error of the means. ^2^ Calf weaning weight is from the grand dam of the heifer. ^3^ Actual calf weaning wt./cow wt. at weaning. ^4^ 6/7 indicates 6- and 7-yr old cows. ^5^ 8+ indicates 8-yr old to 14-yr old cows. ^a,b^ Means within a row lacking a common superscript differ (*p* ≤ 0.05).

**Table 2:** The effect of dam age on subsequent cow production and longevity characteristics in Angus beef females.

	Dam Age, Years							
Category	2	3	4	5	6/7	8+	SE ^1^	*p*-Value
Cow yearling weight, kg	355.23 ^a^	371.96 ^b^	381.51 ^bc^	386.24 ^c^	386.58 ^c^	386.61 ^c^	6.57	<0.01
Present at 5 yr, %	76.70	60.47	70.77	77.36	59.01	72.14	5.32	0.05
Productivity thru 5 yr ^2^, kg	614.02 ^ab^	559.79 ^a^	641.68 ^ab^	727.87 ^ab^	757.15 ^ab^	797.48 ^b^	60.37	0.01

^1^ Pooled standard error of the means. ^2^ Total pounds of calf weaned through 5 years. ^a–c^ Means within a row lacking a common superscript differ (*p* ≤ 0.05).

**Table 3:** The influence of cow age on beef cow production and reproductive measurements for Angus beef females.

	Cow Age, Years							*p*-Value		
Category	2	3	4	5	6/7	8+	SE ^1^	Cow Age	Dam Age	Cow × Dam
Cow BCS at weaning	4.96 ^a^	5.17 ^b^	5.36 ^c^	5.52 ^de^	5.60 ^d^	5.40 ^ce^	0.09	<0.01	0.34	0.19
Cow wt. at weaning, kg	526.62 ^a^	575.95 ^b^	624.30 ^c^	650.74 ^d^	663.51 ^e^	660.61 ^de^	5.54	<0.01	0.04	0.16
Calf birth wt., kg	34.16 ^a^	39.11 ^b^	40.29 ^bc^	41.65 ^cd^	42.50 ^d^	42.21 ^d^	0.59	<0.01	0.19	0.31
Calf 205 d wt., kg	233.72 ^a^	257.32 ^b^	268.92 ^c^	272.28 ^cd^	279.65 ^e^	276.91 ^de^	5.16	<0.01	0.03	0.29
Calf weaning wt., kg	223.72 ^a^	243.51 ^b^	253.59 ^c^	259.13 ^cd^	262.92 ^d^	261.48 ^d^	4.89	<0.01	0.24	0.91
Calf Julian birth date	78.91	81.34	81.31	79.93	82.46	81.65	1.69	0.12	0.49	0.81
WWR ^2^, %	41.19	41.92	41.45	40.56	40.64	40.32	0.94	0.56	0.10	0.63
Pregnancy status, %	90.42	89.31	89.24	86.40	88.91	85.28	1.70	0.44	0.08	0.45
AI conception, %	60.80	59.98	64.46	61.70	64.79	61.87	2.91	0.98	0.85	0.98

^1^ Pooled standard error of the means. ^2^ WWR = actual calf weaning wt./cow wt. at weaning. ^a–e^ Means within a row lacking a common superscript differ (*p* ≤ 0.05).

## Data Availability

The data used in this manuscript are available in the Supplementary Tables S1 and S2.

## Supplementary Materials

The following supporting information can be downloaded at: https://www.mdpi.com/article/10.3390/ani12202768/s1, Table S1: includes the number of observations for each year by cow and dam age; Table S2: includes the lifetime productivity data.
